# The relationship between motor proficiency and reading ability in Year 1 children: a cross-sectional study

**DOI:** 10.1186/s12887-018-1262-0

**Published:** 2018-09-05

**Authors:** N. Milne, K. Cacciotti, K. Davies, R. Orr

**Affiliations:** 1 Physiotherapy Department, Health Sciences and Medicine, Bond Institute of Health and Sport, Bond University, Robina, QLD 4226 Australia; 2 Queensland Government, Gold Coast, Australia

**Keywords:** Physical activity, Curriculum, Exercise, Motor skills

## Abstract

**Background:**

Movement and physical activity is crucial to brain development and has a positive impact on the ability to learn. With children spending a large portion of their time in the school setting, physical activity and the development of motor skills in this environment may not only impact their overall development but may also influence their learning. The aim of this study was to investigate relationships between motor proficiency and reading skills in Year-1 children.

**Methods:**

A cross-sectional study with a single class of Year-1 students (*n* = 24: mean age = 6.07 ± 0.35 years). Assessments included; a) Process Assessment of the Learner (PAL-II) – Diagnostics for Reading and Writing (reading components only); b) Bruininks-Oseretsky-Test-of-Motor-Proficiency (BOT2); c) parent-reported height/weight and; d) Preparatory Year academic reports. The PAL-II was individually administered. The BOT2 was administered in small groups. Parent-reported height and weight measurements as well as Preparatory Year reports provided by the school Principal were obtained for each participant.

**Results:**

Significant negative relationships were obtained between Year-1 children’s total motor proficiency and silent reading ability (*r* = −.53 to −.59, *p* ≤ .01). While not significant for female students, the relationships were significant and strongly correlated for male students (*r* = −.738 to −.810, *p* ≤ .001). Children with low-average English grades demonstrated a strong positive relationship between motor proficiency and pre-reading skills, essential to functional reading (*r* = .664., *p* = .04 to *r* = .716, *p* = .04).

**Conclusion:**

For children with low-average English grades, the strong, positive relationship between motor proficiency and pre-reading skills suggests that this population may benefit from additional motor proficiency skills. Blending of motor skills within the English curriculum may benefit both of these sub-groups within a classroom environment.

## Background

Children spend a large portion of their time in the school setting; an environment that not only influences their learning, but impacts their overall development [1]. Movement and physical activity is not only crucial to brain development but it has a positive impact on the ability to learn [2–6]. Furthermore, exercise facilitates a child’s executive functioning (selecting, organising and properly initiating goal-directed actions) which is important for academic achievement [7]. Research regarding developmental movement programmes; commonly implemented in early childhood curriculum, has also established that movement enhances academic outcomes, specifically in reading and mathematical skills [8]. Likewise, students who enter school with co-morbid movement-related presentations, such as developmental coordination disorder (DCD), have been found to present with both motor and early academic difficulties [9]. Students with DCD are also more likely to demonstrate poor academic outcomes as teenagers [9]. The above-mentioned studies suggest that a curriculum which focusses on a child’s physical activity (underpinned by fine and gross motor skills) may be associated with enhanced neurodevelopment relevant to learning and therefore improve academic achievement.

There is a growing body of evidence demonstrating a relationship between fine motor proficiency and reading ability [10–13]. Additionally, there are positive associations between physical activity and increased academic performance [7–9], however, there is minimal research available on the link between gross motor proficiency, which underpins physical activity participation [14], and reading abilities. Investigating the link between children’s motor proficiency and reading ability may provide clarity around the key factors contributing to the relationship between physical activity and academic outcomes. This information could then be used in planning classroom activities to achieve optimal academic outcomes for children within the school environment. Therefore, the aims of this study were to; i) investigate the relationship between motor proficiency (fine and gross) and reading skills in Year 1 students and; ii) investigate if the relationship differed between male and female students or between those with high–very high English curriculum grades compared to those with average-low English curriculum grades.

## Methods

### Participants

The study sample consisted of a cohort of Year 1 students (*n* = 24: female *n* = 11, male *n* = 13) aged 5 to 7 years (mean age = 6.07 ± 0.35 years) recruited from a primary school in Queensland, Australia with the school selected opportunistically. The selection of the Year 1 cohort was based on discussions and guidance from the school Principal as well as the classroom teacher’s willingness to participate with the study. Informed consent was gained from parents/carers of the child participants after they had read an explanatory statement and had the opportunity to attend an information session about the study during the first week of the school term.

All children from a single Year 1 class were initially invited to participate. Consent was not gained for three students in the class and participation was therefore offered to children in the adjoining Year 1 class, where consent was gained for an additional three children to participate. The research protocol was approved by Bond University Human Research Ethics Committee (RO-1760) with research approval granted by the Department of Education, Training and Employment, Queensland Government. All research was conducted in accordance with the Declaration of Helsinki (1964).

### Outcome measures

The following measures were collected for each of the children in the study: i) end-of-preparatory-year academic reports; ii) parent reported height and weight measurements; iii) Process Assessment of the Learner (PAL-II) – Diagnostics for Reading and Writing scores (reading components only) [15] and; iv) Bruininks Oseretsky Test of Motor Proficiency, 2nd Edition (BOT2) [16] assessments. The Preparatory Year (being the year completed prior to Year 1) reports were provided by the school principal during the first week of the study. These were collected in order to characterise English curriculum academic ability of the participants. In addition, non-identifiable curriculum reports for all Preparatory Year classes (6 classes) in the participating school were provided, to allow the research team to determine if the study group was representative of a typical Year 1 class for English grades. Height and weight measurements were taken at home by parents or guardians and documented on a student database file with the students past medical record. These anthropometric measures were compared to normative data for Australian children. The student database file was returned to researchers in a sealed envelope. The PAL-II and BOT2 testing was performed during class time in the second week of the first school term in Year 1. These assessments were conducted over a one-week period by two qualified physiotherapists with paediatric experience and a trained physiotherapy student.

The PAL-II is an individually administered, norm-referenced, set of measures designed to assess the development of reading and writing processes in children in Kindergarten through to Grade 6 [15]. The PAL-II consists of subtests assessing processes and skills relevant to reading and writing acquisition, including phonological processing, orthographic processing, and rapid naming. The PAL-II has been test reviewed, and although the range of reliability coefficients is large, most of the subtests have displayed good test-retest reliability coefficients [17]. The PAL-II was therefore used to evaluate the reading skills of the Year 1 students in the study. The PAL-II was piloted on two subjects, external to the study cohort, by the research team to determine which sections were most relevant for the study, taking into account the time limitations in the classroom environment. Each child was tested individually, in a quiet, distraction free environment. To appropriately test and represent English literacy / reading skills, the following PAL-II sub sections were used: Phonological decoding, morphological decoding, silent reading (sentence sense) and phonological coding. Phonological decoding involves the examinee reading a list of made-up words (Pseudoword decoding subtest) and has a visual trigger, requiring an oral motor output. Morphological decoding involves the examinee reading a list of related words and has a visual trigger requiring an oral motor output. Silent reading is tested with ‘sentence sense’ and involves the examinee choosing the correct sentence out of three options, two of which have errors. The sentence sense subtests therefore have a visual trigger but requires the child to understand the content in order to determine which sentence is correctly formed before using a motor output (pointing) to respond. Finally, phonological coding was examined using three subtests: i) syllables - where the examinee says a word, then repeats it with a syllable taken out; ii) phonemes - in which the examinee says a word, then repeats it with a phoneme taken out; and iii) rimes - in which the examinee says a word, then repeats it with the rime taken out. These subtests therefore offer an auditory trigger with an oral motor output and are combined to make up the Phonological Coding composite score. Higher composite scores indicate a higher performance for each of the subtests.

The BOT2 is a valid and reliable norm-referenced diagnostic measure of motor proficiency commonly used by physiotherapists and occupational therapists in clinical and school practice settings [16]. It is an individually administered measure of motor proficiency (including fine and gross motor skills) of children and youth aged four through 21 years. It is intended for use by practitioners and researchers as a discriminative and evaluative measure to characterise motor performance [16]. In the BOT2, Fine Motor Precision and Fine Motor Integration subtests combine to make up *Fine Manual Control.* Manual Dexterity and Upper Limb Coordination subtests combine to make up *Manual Coordination.* Bilateral Coordination and Balance subtests combine to make up *Body Coordination.* Running Speed and Agility and Strength combine to make up *Strength and Agility.* The sum of *Fine Manual Control, Manual Coordination, Body Coordination and Strength and Agility* composite scores, make up *Total Motor Proficiency*. All BOT2 total point scores were scaled for age and gender. A high score on the BOT2 indicates a high motor proficiency with composite scores over 70 indicating well-above average motor ability and under 30 indicating well-below average ability. BOT2 percentiles have been reported as the indication of motor proficiency in this study. The BOT2 was administered according to test instructions with the exception of students working in small groups (2–4 students) which assisted with reducing the time spent out of class.

### Statistical analysis

The end of semester reports from the Preparatory Year were originally scored on a five-point scale, as per the school’s assessment framework, using the following categories from lowest to highest: (1) Becoming Aware, (2) Exploring, (3) Working With, (4) Making Connections and (5) Applying. For the purpose of this study, these narrative descriptive grade categories were converted to be consistent with commonly understood language and divided into two groups: Group 1 consisted of ‘low’ to ‘average’ English curriculum results (Categories 1–3) and Group 2 of ‘high’ to ‘very high’ English curriculum results (Categories 4–5). Data analysis was performed for Year 1 children as a whole cohort and with participants divided into groups based on gender and then English curriculum results.

A chi-squared test for goodness of fit was conducted to determine if distributions of English curriculum grades for study participants were representative of those of the entire Year 1 population at the study school. Independent samples t-tests were used to determine if differences in the mean scores existed between groups (i.e. children with low-to-average English grades compared to children with high-to-very high English grades and males compared to females). The assumption of homogeneity of variances was assessed using Levene’s test prior to analysis with unequal variances accommodated for where identified. Pearson’s product moment correlations were used to determine relationships between reading skills (PAL-II) and motor skills (BOT2) for the study cohort as a whole and for individual subgroups (gender and English grades). Narrative descriptions regarding the strength of relationships for Pearson’s correlations were applied using criteria previously reported by Evans [18]. To investigate whether differences between English grades correlations existed, a Fisher r-to-z transformation, which calculated a value of z, was performed. Alpha levels were set at 0.05 a priori. Data for this study remains stored on a locked password protected file in the organisation approving the study protocol. De-identified data may be made available on request with authorisation from the relevant organisations and research / ethics committee.

## Results

### Participants

From the 24 student participants (female *n* = 11, 46%, mean age = 5.95 ± 0.28, range 5 to 7 years: male *n* = 13, 54%, 6.13 ± 0.36, range 5 to 7 years) who consented to participate in the study, one male student was excluded from analysis due to having incomplete data with his Preparatory Year report being inaccessible. Characteristics of study participants, as a total group (*n* = 23) and by sub groups (gender and English grades), are provided in Table 1. Parents / guardians of five Year 1 participants did not complete either the parent reported height and/or weight measurement section on the parent database resulting in only 18 BMI datasets for analysis. Two children were reported to have a past history of an acquired brain injury. Both were reported by their parents to be typically developing at the time of the study; one child displayed below average motor skills (within 1 SD of the norm reference for their age and gender) and one child displayed above average motor skills (2 SD from the norm reference for their age and gender). A sensitivity analysis revealed that neither of these children presented as outliers within the study population for motor or reading skills. One student did present as an outlier in a pre-reading subtest (Morphological decoding – accuracy). This outlier was only present during this single subset of data and as such was not removed from overall analysis, however, where a potential impact of this outlier on findings existed in a sensitivity analysis this was reported in the results. The study cohort was representative of a typical Australian Year 1 population for age [19] and BMI percentile [19]. The mean motor proficiency (BOT2 percentile rank) was in the average range after scaling for age and gender [16] (see Table 1). Additionally, the participants in the present study where found to be representative of a typical Year 1 cohort in the school for English curriculum grades, exhibiting a wide spread of academic Preparatory Year grade levels and demonstrating no significant difference in English grades when compared to the rest of the school’s Year 1 population (χ2(4) = 2.481, *p* = .648).

**Table 1:** Characteristics of study participants as a Year 1 class cohort and by subgroups

	Class Cohort		Female		Male		Difference *p*-value	Low-Average English grades		High-Very High English grades		Difference *p*-value
	N	Mean ± SD	N	Mean ± SD	N	Mean ± SD		N	Mean ± SD	N	Mean ± SD	
Exact Age (yrs)	23	6.04 ± 0.33	11	5.95 ± 0.28	12	6.13 ± 0.36	0.194	10	5.90 ± 0.31	13	6.15 ± 0.32	0.066
Height (cm)	19	116.76 ± 6.06	7	113.36 ± 5.39	12	118.7 5 ± 5.71	0.060	9	115.70 ± 6.04	10	118.00 ± 6.16	0.289
Weight (kg)	20	21.75 ± 4.14	9	20.30 ± 2.35	11	22.94 ± 4.98	0.162	10	19.82 ± 2.00	10	23.68 ± 4.89	0.033
BMI (%ile)	18	56.11 ± 37.18	7	57.57 ± 40.32	11	55.18 ± 37.04	0.899	9	42.20 ± 31.40	9	70.00 ± 38.95	0.115
Total Motor (%ile)	23	50.74 ± 29.80	11	33.27 ± 26.10	12	66.75 ± 23.92	0.004	10	51.50 ± 35.79	13	50.15 ± 25.82	0.917

Mean PAL-II reading and BOT2 motor proficiency scores are presented by cohort, as well as by subgroups (gender and English grades) in Table 2. Independent samples t tests revealed no significant differences between male and female participants for pre-reading skills (see Table 2). However, male participants in this study presented with significantly lower sentence sense accuracy and fluency (i.e. lower in the more advanced reading skill assessments). In addition, higher total motor proficiency scores were found in male participants when compared to female participants (*p* < 0.05) even after the BOT2 scoring had accounted for expected gender differences by scaling the raw data for age and gender (see Table 2). A detailed analysis of motor proficiency subtests suggests that this is largely attributable to the male participants having significantly better manual coordination (including ball skills) and body coordination than the female participants (see Table 2). When comparing the study cohort by English grades, independent samples t tests revealed that the High-Very high group performed significantly better than the Low-Average group in all reading skill subtests of the PAL-II, except in those subtests that required the participant to read and discriminate correct full sentences (i.e. Sentence Sense) (see Table 2). There were no significant differences in motor proficiency between these two groups.

**Table 2:** Mean PAL-II reading scores and BOT2 motor proficiency scores by cohort and subgroups

Variable (Percentile)	Class Cohort		Female		Male		Difference between gender	Low-Average English grades		High-Very High English grades		Difference between English grade groups
	N	Mean ± SD	N	Mean ± SD	N	Mean ± SD	*p*-value	N	Mean ± SD	N	Mean ± SD	*p*-value
PAL-II												
*Phonological Decoding (PDF60ile – Fluency)*	23	41.57 ± 23.07	11	39.7 3 ± 27.27	12	43.25 ± 19.54	0.724	10	26.60 ± 12.63	13	53.08 ± 22.93	0.004**
*Phonological Decoding (PDAile - Accuracy)*	23	43.48 ± 26.80	11	45.27 ± 30.72	12	41.83 ± 23.93	0.766	10	24.10 ± 9.60	13	58.39 ± 26.35	0.001**≠
*Morphological Decoding (MDFAile - Accuracy)*	23	29.87 ± 24.32	11	27.91 ± 28.23	12	31.67 ± 21.25	0.720	10	12.30 ± 9.92	13	43.39 ± 23.57	0.001**≠
*Morphological Decoding (MDFile - Fluency)*	23	16.04 ± 19.71	11	12.44 ± 19.70	12	19.33 ± 19.98	0.414	10	4.18 ± 4.97	13	25.15 ± 22.05	0.005**≠
Sentence Sense Accuracy *(SSAile)*	23	19.57 ± 23.50	11	31.55 ± 29.38	12	8.58 ± 6.65	0.028*†≠*	10	13.70 ± 16.90	13	24.07 ± 27.34	0.305
Sentence Sense Fluency *(SSFile)*	23	19.70 ± 22.61	11	30.27 ± 28.48	12	10.00 ± 8.40	0.043*†≠*	10	13.50 ± 14.83	13	24.46 ± 26.75	0.258
*Phonological Coding Composite (PLCile) (Syllables, Phonemes, Rimes)*	23	26.12 ± 19.77	11	23.71 ± 24.14	12	28.33 ± 15.53	0.587	10	16.54 ± 14.13	13	33.20 ± 20.78	0.038*
BOT2												
Fine Manual Control	23	49.70 ± 25.21	11	42.27 ± 26.57	12	56.50 ± 22.88	0.182	10	43.20 ± 29.38	13	54.69 ± 21.34	0.289
Manual Coordination	23	40.61 ± 28.70	11	22.55 ± 24.25	12	57.17 ± 22.07	0.002*†*	10	47.90 ± 31.74	13	35.00 ± 26.00	0.296
Body Coordination	23	36.48 ± 25.91	11	21.55 ± 16.51	12	50.17 ± 25.85	0.005*†*	10	32.40 ± 28.62	13	39.61 ± 24.33	0.521
Strength and Agility	23	71.65 ± 27.19	11	62.73 ± 28.23	12	79.83 ± 24.52	0.135	10	74.80 ± 25.65	13	69.23 ± 29.11	0.637
Total Motor Proficiency	23	50.74 ± 29.80	11	33.27 ± 26.10	12	66.75 ± 23.92	0.004*†*	10	51.50 ± 35.79	13	50.14 ± 25.82	0.917

† There is a significant difference between genders at *p* < .05:

*There is a significant difference between academic groups at *p* < .05:

**There is a significant difference between academic groups at *p* = <.01

≠ Equal variances are not assumed

Pearson’s correlations between motor proficiency (BOT2) and reading skills (PAL-II) by cohort, gender and English grade subgroups are provided in Table 3. For the class cohort there was a significant moderate negative relationship between Total Motor proficiency and Sentence Sense (accuracy and fluency). A similar significant negative relationship between each of the gross motor related subtests (Manual Coordination, Body Coordination and Strength and Agility) and Sentence Sense (accuracy and fluency) was present (see Table 3). The relationship between Total Motor Proficiency and Sentence Sense (fluency and accuracy) was diminished when examined in females alone but strengthened considerably in male participants who were noted to have significantly higher mean motor proficiency scores (*p* = < 0.05). For children with Low to Average English grades, all relationships between Motor Proficiency and Reading subtests (other than the relationship between Total Motor Proficiency and Sentence Sense) were positive, with strong significant correlations noted between Total Motor Proficiency and; Phonological Decoding (Fluency and Accuracy) and Morphological Decoding (Accuracy) (see Table 3). However, with the outlier removed Morphological Decoding (Accuracy) was no longer significantly related to Total Motor Proficiency (*p* = 0.052). Table 4 demonstrates that the relationships between all pre-reading subtests (Phonological Decoding, Morphological Decoding and Phonological Coding) and Total Motor proficiency were significantly different between children in the Low-Average English grades group compared to those in the High-Very High English grades group. No significant differences were found in the relationship between Total Motor Proficiency and Sentence Sense in children with High – Very High English grades compared to those with Low-Average English grades (see Table 4).

**Table 3:** Pearson’s correlations between motor proficiency and reading skills by gender and English grades

Reading Skills (PAL-II Subtests)	Motor Proficiency (BOT2 Percentile Ranks)				
	Fine Manual Control r (*p*-value)	Manual Coordination r (*p*-value)	Body Coordination r (*p*-value)	Strength and Agility r (*p*-value)	Total Motor r (*p*-value)
Class Cohort (*n* = 23)					
*Phonological Decoding (PDF60ile – Fluency)*	0.01 (0.96)	−0.02 (0.95)	− 0.09 (0.69)	0.11 (0.61)	0.01 (0.98)
*Phonological Decoding (PDAile - Accuracy)*	0.05 (0.82)	−0.08 (0.72)	−0.21 (0.35)	0.112 (0.61)	−0.05 (0.82)
*Morphological Decoding (MDFAile - Accuracy)*	−0.03 (0.89)	− 0.10 (0.65)	− 0.04 (0.84)	0.05 (0.83)	− 0.05 (0.83)
*Morphological Decoding (MDFile - Fluency)*	− 0.17 (0.45)	− 0.13 (0.57)	− 0.075 (0.74)	− 0.315 (0.14)	− 0.23 (0.29)
Sentence Sense Accuracy *(SSAile)*	−0.18 (0.41)	− 0.44** (0.04)	− 0.45** (0.03)	− 0.53** (0.01)	− 0.53** (0.01)
Sentence Sense Fluency *(SSFile)*	−0.22 (0.32)	− 0.49* (0.02)	− 0.48* (0.02)	− 0.61** (< 0.01)	− 0.59**(< 0.01)
*Phonological Coding Composite (PLCile) (Syllables, Phonemes, Rimes)*	− 0.03 (0.88)	− 0.09 (0.68)	− 0.03 (0.88)	− 0.20 (0.37)	− 0.12 (0.59)
Females (*n* = 11)					
*Phonological Decoding (PDF60ile – Fluency)*	0.264 (0.433)	0.046 (0.893)	−0.097 (0.777)	0.384 (0.244)	0.195 (0.566)
*Phonological Decoding (PDAile - Accuracy)*	0.432 (0.184)	−0.055 (0.872)	−0.139 (0.684)	0.335 (0.314)	0.172 (0.612)
*Morphological Decoding (MDFAile - Accuracy)*	0.233 (0.491)	−0.125 (0.715)	−0.169 (0.620)	0.211 (0.534)	0.025 (0.942)
*Morphological Decoding (MDFile - Fluency)*	0.091 (0.790)	−0.075 (0.827)	−0.245 (0.468)	− 0.079 (0.817)	−0.130 (0.702)
Sentence Sense Accuracy *(SSAile)*	0.041 (0.904)	−0.216 (0.524)	−0.278 (0.408)	− 0.478 (0.137)	− 0.328 (0.325)
Sentence Sense Fluency*(SSFile)*	− 0.012 (0.972)	− 0.330 (0.322)	− 0.382 (0.246)	− 0.525 (0.098)	− 0.420 (0.199)
*Phonological Coding Composite (PLCile) (Syllables, Phonemes, Rimes)*	0.249 (0.460)	− 0.078 (0.820)	− 0.184 (0.588)	− 0.147 (0.665)	−0.070 (0.838)
Males (*n* = 12)					
*Phonological Decoding (PDF60ile – Fluency)*	−0.420 (0.174)	−0.257 (0.420)	− 0.237 (0.458)	−0.330 (0.295)	− 0.385 (0.217)
*Phonological Decoding (PDAile - Accuracy)*	−0.414 (0.181)	− 0.043 (0.893)	−0.277 (0.384)	− 0.119 (0.713)	−0.254 (0.426)
*Morphological Decoding (MDFAile - Accuracy)*	−0.459 (0.133)	−0.274 (0.389)	− 0.073 (0.821)	−0.239 (0.454)	− 0.301 (0.342)
*Morphological Decoding (MDFile - Fluency)*	−0.565 (0.056)	− 0.532 (0.075)	−0.207 (0.518)	− 0.734^**^ (0.007)	−0.692^*^ (0.013)
Sentence Sense Accuracy *(SSAile)*	−0.473 (0.120)	−0.265 (0.405)	− 0.488 (0.107)	−0.704^*^ (0.011)	− 0.738^**^ (0.006)
Sentence Sense Fluency*(SSFile)*	−0.474 (0.119)	− 0.315 (0.318)	−0.428 (0.165)	−.916^**^ (0.000)	−.810^**^ (0.001)
*Phonological Coding Composite (PLCile) (Syllables, Phonemes, Rimes)*	−0.599^*^ (0.039)	− 0.426 (0.167)	− 0.093 (0.774)	− 0.427 (0.166)	− 0.487 (0.108)
High – Very High English Grades (*n* = 13)					
*Phonological Decoding (PDF60ile – Fluency)*	−0.660^*^ (0.01)	− 0.067 (0.83)	− 0.594^*^ (0.03)	0.049 (0.87)	− 0.370 (0.21)
*Phonological Decoding (PDAile - Accuracy)*	− 0.437 (0.14)	− 0.079 (0.80)	−0.753^**^ (< 0.01)	0.150 (0.62)	− 0.329 (0.22)
*Morphological Decoding (MDFAile - Accuracy)*	−0.614^*^ (0.03)	− 0.112 (0.72)	− 0.552 (0.05)	0.034 (0.91)	− 0.364 (0.22)
*Morphological Decoding (MDFile - Fluency)*	− 0.634^*^ (0.02)	− 0.084 (0.79)	−0.382 (0.20)	− 0.465 (0.11)	−0.524 (0.07)
Sentence Sense Accuracy *(SSAile)*	−0.092 (0.76)	−0.425 (0.15)	− 0.579^*^ (0.04)	−0.417 (0.16)	− 0.596^*^ (0.03)
Sentence Sense Fluency *(SSFile)*	−0.165 (0.59)	− 0.445 (0.13)	−0.590^*^ (0.03)	− 0.538 (0.06)	−0.682^*^ (0.01)
*Phonological Coding Composite (PLCile) (Syllables, Phonemes, Rimes)*	−0.455 (0.12)	−0.210 (0.49)	− 0.381 (0.20)	−0.330 (0.27)	− 0.473 (0.10)
Low – Average English Grades (*n* = 10)					
*Phonological Decoding (PDF60ile – Fluency)*	0.68^*^ (0.03)	0.61 (0.06)	0.53 (0.12)	0.69^*^ (0.03)	0.716^*^ (0.02)
*Phonological Decoding (PDAile - Accuracy)*	0.58^*^ (0.08)	0.66^*^ (0.04)	0.50 (0.14)	0.68^*^ (0.03)	0.670^*^ (0.03)
*Morphological Decoding (MDFAile - Accuracy)*	0.49 (0.15)	0.55 (0.10)	0.75^*^ (0.01)	0.60 (0.07)	0.664^*^ (0.04)
*Morphological Decoding (MDFile - Fluency)*	0.318 (0.37)	0.378 (0.28)	0.594 (0.07)	0.453 (0.19)	0.473 (0.17)
Sentence Sense Accuracy *(SSAile)*	−0.531 (0.11)	−0.444 (0.20)	−0.424 (0.22)	− 0.815^**^ (0.004)	−0.568 (0.09)
Sentence Sense Fluency *(SSFile)*	−0.594 (0.07)	−0.575 (0.08)	− 0.521 (0.12)	−0.849^**^ (0.002)	− 0.643^*^ (0.045)
*Phonological Coding Composite (PLCile) (Syllables, Phonemes, Rimes)*	0.254 (0.48)	0.360 (0.31)	0.340 (0.38)	0.181 (0.62)	0.359 (0.31)

r: Pearson’s product moment correlations

Correlation is significant at:*p* = < 0.05*, *p* = < 0.01**

**Table 4:** Fisher r-to-z test differences between correlations based on Preparatory Year English grades represented as z scores

Reading Skills (PAL-II Subtests)	Motor Proficiency (BOT2 Percentile Ranks)				
	Fine Manual Control	Manual Coordination	Body Coordination	Strength and Agility	Total Motor
*Phonological Decoding (PDF60ile – Fluency)*	−3.29*†*	−1.57	−1.32	−1.62	−2.61**
*Phonological Decoding (PDAile - Accuracy)*	−2.29*	−1.77	−3.1**	− 1.38	− 2.34**
*Morphological Decoding (MDFAile - Accuracy)*	− 2.54†	− 1.48	−3.23†	− 1.34	− 2.40**
*Morphological Decoding (MDFile - Fluency)*	− 2.19^*^	− 0.98	−2.79**	− 2.10*	−2.22*
Sentence Sense Accuracy *(SSAile)*	1.01	0.05	− 0.42	1.42	−.0.09
Sentence Sense Fluency *(SSFile)*	1.05	0.36	−0.2	1.32	−0.14
*Phonological Coding Composite (PLCile) (Syllables, Phonemes, Rimes)*	−1.52	− 1.2	−1.53	− 1.53	−1.81

Significant difference in correlations (2-tailed) between Low-Average English grades group compared to High-Very High English grades group at:*p* = < 0.05*, *p* < 0.01**, *p* < 0.001†

## Discussion

The main purpose of this study was to investigate the relationship between motor proficiency and reading ability in Year 1 students. Specifically, the first of our study aims was to investigate the relationship between the BOT2 motor proficiency percentiles and the PAL-II reading subtest percentiles in Year 1 students. The findings from the present study suggest that a significant and moderate negative relationship exists between Year 1 student boy’s Total Motor Proficiency (inclusive of fine and gross motor skills) and their ability to read silently with accuracy and fluency (sentence sense). This relationship between motor proficiency and reading was also consistent with that found between the individual gross motor subtests of: Manual Coordination; Body Coordination and; Strength and Agility, and silent reading. However, there were no significant relationships identified between Fine Manual Control (predominately fine motor skills) and reading skills. These results indicated that, in general, Year 1 boys with higher gross motor proficiency were likely to perform more poorly in reading ability and conversely those who performed more poorly in gross motor skills were likely to have higher silent reading ability.

This negative relationship between high gross motor proficiency and poor reading ability could be explained by many factors which are likely to be driven by children’s personal preferences and self-competency beliefs, the most obvious of which is likely to be the time spent being physically active and developing motor skills [20] or the time spent reading [21]. Indeed, previous research has demonstrated a strong positive association between the time a student spends on homework with academic achievement; with those students who spend more time doing homework tending to obtain higher academic grade results, which supports this possible explanation [22]. This explanation is also supported by the results of the present study, which show that the male students who participated in this study had significantly higher motor proficiency than the female students, particularly in Manual and Body Coordination (see Table 2). When the results were separated by gender, the negative relationship between motor proficiency and silent reading for the female students was lower and did not reach significance. Conversely for the male students this relationship was not only significant but strongly to very strongly negatively correlated (see Table 3).

Considering these findings, the male students also presented with significantly lower mean silent reading scores (see Table 2) when compared to the female students (Sentence Sense Accuracy: *p* = .028 and Sentence Sense Fluency: *p* = .043). As such the disparity between motor proficiency and silent reading was notably greater in the male students (see Table 3). These findings are consistent with previous literature which suggests that young male readers are lagging behind their female counterparts in literacy skills, taking longer to learn how to read but also reading less and valuing reading less than females [23]. This discrepancy between males and females for early reading development has led to a number of texts outlining the importance of meeting the interests of young males both in the content and the mode of delivery for reading. *Reading Don’t Fix No Chevys: Literacy in the Lives of Young Men* [24] provides an example of this push for pedagogical enhancement for young males to learn lifelong reading skills. Furthermore, when surveyed around the objection to reading, males stated four resounding arguments: the activity was boring/no fun, they had no time/were too busy, they preferred other activities over it, they weren’t able to get into stories and they were not good at it [25]. These findings and the associated literature suggest that there is an opportunity to modify the reading curriculum to be more physically active, to better engage young males in the learning process of reading.

Interestingly for the study cohort as a whole class, no significant relationships were identified between motor proficiency and the reading subtests of Phonological Decoding, Morphological Decoding and Phonological Coding (see Table 3), which are important skills for developing functional reading competency [26–31]. The Phonological Decoding and Morphological Decoding subtests of the PAL-II require a visual stimulus and an oral motor output, without the need to demonstrate comprehension of the individual words presented. Conversely, the Sentence Sense (silent reading) subtests have a visual trigger but require the child to read three full similar sentences, not just individual words and comprehend the information to the extent that they can identify errors in a full sentence. Evidently this task is a higher-level reading task and requires more cognitive processing (i.e. comprehension) than the other reading subtests, so it may be that the relationship between motor proficiency and silent reading is influenced by general cognitive development and academic ability and not just reading and motor skills. Further research is therefore warranted in this field to provide additional insights into this relationship.

To further address the second of our study aims, we subdivided the class cohort and examined the relationship between motor proficiency and reading for children with High-Very High English grades compared to children with Low-Average English grades. We found significant differences in the correlations between motor proficiency and a number of pre-reading skills for children in these two groups (see Table 4). Perhaps the most intriguing results of this study are the significant and strong positive relationships found between Total Motor proficiency and the pre-reading skills (Phonological Decoding and Morphological Decoding) of children with Low–Average English grades compared to the non-significant relationships between these variables in children with High – Very High English grades. The relationship between Total Motor proficiency and pre-reading skills was significantly different between these two groups (see Table 4). It is apparent that to develop competent reading, as indicated by the more advanced silent reading (Sentence Sense) subtest, children must first become competent in the pre-reading subskills (i.e. Phonological Decoding, Morphological Decoding and Phonological Coding). The fact that in children with Low-Average English grades, a significant positive relationship exists between these pre-reading skills which have a visual trigger with an oral motor output, and Total Motor proficiency, may mean that these children use their motor skills to assist with learning the pre-reading skills (i.e. pointing to track letters and words, coordination to help with sequencing of sounds / phonemes and consolidating the learning process of syllables through activities such as clapping / stomping etc.) and perhaps the influence of motor proficiency on reading ability may decline once a threshold of pre-reading skills are achieved. This emerging bell curve is not atypical in areas of human performance and has been noted in in works by Yerkes-Dodson [32] in relation to performance. It is possible that after children develop their foundation pre-reading skills, other factors may mediate the relationship between motor proficiency and functional reading (Sentence Sense) (i.e. time spent practicing reading, cognition, physical activity or fitness). For example, if a child has already learned to read functionally, the relationship between motor skills and reading may be more strongly influenced by time spent practicing reading or practicing motor skills. Further research is warranted to investigate this trend with larger year 1 cohorts.

Alternatively, it is possible that the relationship between motor proficiency and reading skills will remain different between these two groups of children for years to come even after acquiring functional reading skills. The employment of different learning styles for developing reading skills, may vary the rate that a child learns to read functionally. This possible explanation for our study findings, is consistent with the varying styles for learning commonly presented in the educational literature. For example, some children may use a kinaesthetic learning style (employing motor skills) to develop basic reading proficiency whereas other children may develop reading skills through their visual and or auditory systems without needing to employ motor skills. It is beyond the scope of this study to examine the causal effects on reading ability, however the results of the present study suggest that further research exploring the impact of enhancing motor proficiency on reading outcomes for children who, when expected to, have not yet mastered functional reading is warranted.

Another important finding from this study was the persistent negative relationship between motor proficiency and the most advanced of the reading subtests (sentence sense – silent reading) irrespective of the children’s English grades. This could be attributed to the timing of data collection, being the first week of Term 1 for the school year. The PAL II was standardised for Year 1 children yet the mean score for these two subtests was below the 25th percentile irrespective of their previous Preparatory Year English grades. As the PAL II could only be scaled for Year 1, not age by months, it is possible that if the PAL II was administrated again later in the school year, many children would have developed higher functional reading skills (i.e. higher sentence sense scores) and the relationship with motor proficiency may then have moved towards a positive correlation at the end of Year 1. Further longitudinal research using the same measures, is needed to explore this phenomenon more closely over time.

It is important to acknowledge that there are a number of limitations to this study. The small number of participants (*n* = 24) was the most noteworthy limitation in this study. Furthermore, in order to achieve the second aim of the study, which included investigations of sub groups (by gender and English grades), the sample sizes were reduced and as such, the potential for family-wise error rates given to multiple correlations with the smaller sample sizes exists. Considering this, the sub group investigations were retained in order to at least inform potential sub group influences and to guide future research. Although the study class cohort as a whole appeared to be representative of Australian children for motor proficiency, BMI and English grades, there was a significant difference between males and females with males having better gross motor and total motor proficiency, after scoring methods had accounted for age and gender. A larger population that maintains the representativeness of Australian Year 1 children would increase the statistical power and allow for a reduction in potential external bias. Furthermore, our small study cohort and use of multiple correlations increases the likelihood of Type 1 errors in our data and this could be prevented in future research by including larger study populations. A class inclusion approach was also a limitation as the inclusion of two students who had a history of acquired brain injury may have influenced the results. It should be noted however, that these children were observed to have no obvious residual effects of their brain injuries. Furthermore, the present study did not account for any unidentified learning or movement difficulties that children may have had. Lastly, although the PAL-II demonstrates good test-retest reliability coefficients [17], the selection of the PAL-II, as the reading outcome measure meant that raw results were scaled only for grade (i.e. Grade 1), rather than age, leaving age as a possible contributing factor in the reading outcomes of children. Much research regarding reading skills and fluency has been undertaken to develop normative data for use in reading assessment and screening tools, suggesting that reading should be screened and assessed regularly due to the rapid development of reading skills in kindergarten and Year 1 children and to ensure that children do not miss critical milestones for reading [33–37]. Whilst the Year 1 children in our study had a mean age of 6.04 years, there was a standard deviation of approximately 4 months in our study cohort. It is possible that the older children may have developed critical pre-reading milestones compared to the younger children and future research using this tool should consider age as an additional potential confounding variable to be controlled.

## Conclusion

The results of this study demonstrate that in a whole of class cohort of Year 1 students, a significant negative relationship exists between children’s total motor proficiency and their silent reading ability. However, this relationship was only significant in male students and potentially this relationship was enhanced through the notable differences between their higher motor proficiency and lower silent reading scores when compared to female students in our study cohort. On first take, these findings appear to support the notion that for male children in particular, there is a disparity between their motor skill development and their reading skills such that it appears that the more time they spend being physically active and developing motor skills, the poorer their reading skills will be. However, important additional findings of this study are that children with low-average English grades, who may be considered ‘pre-readers’, demonstrated a strong and positive relationship between motor proficiency and pre-reading skills (i.e. Phonological Decoding and Morphological Decoding). Based on these findings we propose that in the early childhood stages when a child is learning to read (i.e. not yet able to accurately and fluently read sentences and is identified with low English grades), offering the integration of motor skills within the English curriculum, may engage students, particularly those with kinaesthetic learning preferences in the pre-reading learning process and enhance the development of pre-reading skills, which are required to eventually progress to functional reading. Furthermore, individual learning preferences of children could be explored where children who demonstrate a strong tendency for using kinaesthetic learning styles could have motor skills further integrated into their pre-reading curriculum. Future research could examine the effect of enhancing motor skill proficiency of pre-readers on children’s future reading ability. This could be done by modifying the reading curriculum for pre-readers to be more motor skills based (e.g. clapping / jumping out syllables or improving hand-eye coordination for visual tracking in reading) to potentially improve their future reading capabilities. This approach may engage those kinaesthetic learners who not do not flourish learning academic curriculum in a desk-top classroom situation and would have the added benefits for all children of potentially enhancing motor skills, leading to greater physical activity and improving health outcomes in addition to academic outcomes [38–40]. Further studies investigating the link between motor proficiency and other academic subjects could also be considered in future research in this field. The results of this study should challenge the policies implemented in schools where English curriculum and specifically early reading curriculum is commonly delivered as mostly desktop-based or sedentary learning activities. As this study has demonstrated that for children with Low – Average English grades, motor proficiency has a strong significant positive association with pre-reading skills such as phonological and morphological decoding which are required for children to become functional readers.

## Abbreviations

BMI: Body mass index
BOT2: Bruininks-Oseretsky-Test-of-Motor-Proficiency
PAL-II: Process Assessment of the Learner
